# Co-Occurrence of Rheumatoid Arthritis and Lung Cancer—Coincidence or Not?

**DOI:** 10.3390/life13102089

**Published:** 2023-10-20

**Authors:** Ioana Munteanu, Constantin Gheorghevici, Catalin Constantin Coca, George Alexandru Diaconu, Alexandra Emilia Sandru, Nicolae Feraru, Andreea Popa, Roxana Nemes, Beatrice Mahler

**Affiliations:** 1 Faculty of Medicine, Titu Maiorescu University, 040441 Bucharest, Romania; ioana.munteanu2015@yahoo.ro (I.M.);; 2 “Marius Nasta” Institute of Pneumophtisyiology, 050159 Bucharest, Romaniabeatrice.mahler@umfcd.ro (B.M.)

**Keywords:** bronchopulmonary cancer, rheumatoid arthritis, methotrexate, biopsy, ultrasound

## Abstract

Bronchopulmonary cancer is the leading cause of cancer deaths globally. Rheumatoid arthritis is one of the risk factors for lung cancer, and those who use methotrexate have a higher risk of developing lung cancer. We present the case of an 80-year-old patient who is a former smoker and is known to have rheumatoid arthritis, being treated using methotrexate; they were brought by ambulance to the emergency room for coughing with ineffective expectoration, dyspnea on slight exertion, and right-lateral chest pain with onset about one month prior and progressive worsening. Imaging showed a 7 cm/6 cm LID tumorous lung formation with parietal invasion and C7 rib lysis, as well as diffuse fibrotic interstitial changes predominantly in the lower lobes. An ultrasound-guided transthoracic lung biopsy was performed, and histopathological examination established the diagnosis of invasive squamous cell lung carcinoma, G2. In conclusion, the chest pain interpreted by the patient as rheumatic pain delayed the diagnosis of lung cancer; the patient presented rather late to the hospital once respiratory failure set in.

## 1. Introduction

Rheumatoid arthritis (RA) is a chronic autoimmune disease characterized by joint inflammation and systemic manifestations. It primarily affects the joints, leading to pain, swelling, and stiffness. However, research has suggested a potential association between RA and an increased risk of developing certain types of pulmonary pathologies, including lung cancer [1]. Other than that, some therapies, including DMARDs, have been shown to increase the risk of developing neoplasms [2], but the results often contradict, and a definite theory has yet to be stipulated. There are multiple risk factors to be considered, some of which cannot be changed (such as genetic susceptibility to neoplasms) or are medium-dependent. The annual incidence of RA is reported to be approximately 40/100,000 people worldwide, with women being three–four times more affected by this chronic inflammatory disease [1].

Bronchopulmonary cancer is a major health problem and the leading cause of cancer deaths globally according to the GLOBOCAN database. It is the second most diagnosed neoplasm in the world after breast cancer [3].

Smoking is the main risk factor for developing bronchopulmonary cancer. Up to 85% of lung cancer cases are due to smoking. Tobacco consumption has also been found to increase the risk of rheumatoid arthritis by 40%. Smoking may therefore be a common risk factor for both diseases [1].

Understanding the link between RA and lung cancer is crucial for prevention, early detection, and effective management of both conditions. This paper will report the case of our patient, N.C., and will explore the existing literature data on the association between rheumatoid arthritis, methotrexate use or dosage, and lung cancer, providing relevant references to support the discussion.

## 2. Materials and Methods

We will discuss the case of our patient, N.C., male, aged 80, diagnosed with severe RA under DMARDs treatment, who recently developed respiratory symptoms. In the quest of finding a diagnosis, the patient’s previous medical records were used and photos of his RA-impaired joints (both hands and feet) were taken; this was all carried out with the patient’s consent. In addition, in the making of this paper, we used imaging, respiratory function tests, histopathological reports, and bloodwork that were performed at the hospital during the admission. The patient had signed informed consent regarding sharing medical data.

## 3. Results

An 80-year-old male, who is an ex-smoker (40 packs/year) and who was previously diagnosed with rheumatoid arthritis (treatment: methotrexate, sulfasalazine, folic acid, Plaquenil, prednisone) was brought by ambulance to the on-call room of the “Marius Nasta” Institute of Pneumophysiology in Bucharest, Romania, for coughing with ineffective expectoration, dyspnea during small efforts, weight loss, and right-lateral thoracalgia with onset of about one month and progressive worsening.

**Figure 1:**
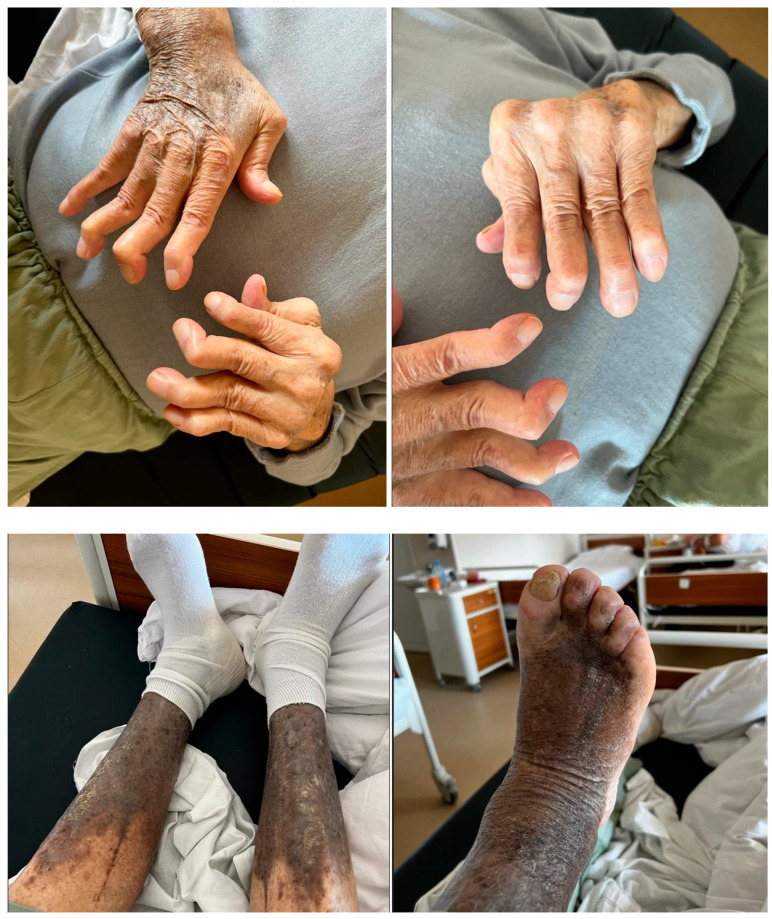
Joint implications of RA and dermatitis.

Objective examination on admission: Altered general condition; conscious; cooperative; afebrile; chest kyphoscoliosis; acoustically vesicular murmur bilaterally present; diffuse bronchial rales bilaterally; SaO_2_ = 83% in ambient air and sitting down corrected to 94% with 4L O_2_/min via oronasal mask; rhythmic heart sounds; no heart murmurs; AV = 90 bpm; BP = 140/100 mmHg; superficial ganglionic system not palpable; poorly represented connective tissue; abdomen painless on palpation; bowel transit present; liver at border; spleen not palpable; normal urination; negative Giordano maneuver; joint deformities of hands and feet; Heberden and Bouchard nodes; ochreous dermatitis of lower limbs and forearms (Figure 1).

**Figure 2:**
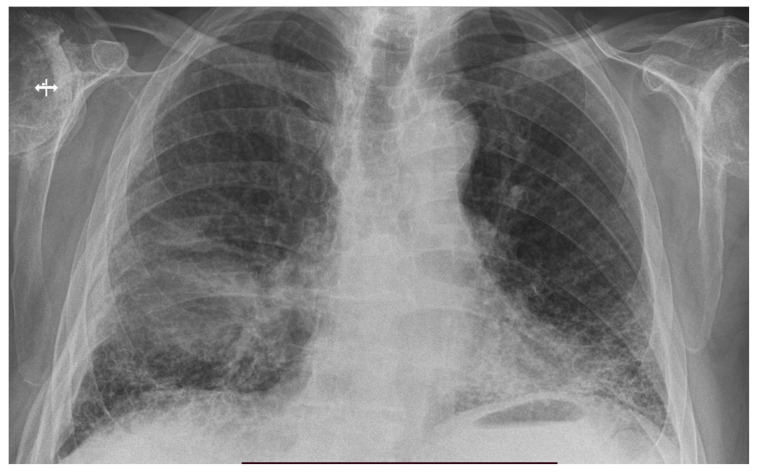
Chest X-ray showing right lower lobe opacity and fibrosis.

On admission, the chest X-ray showed the following: subcostal opacities with irregular borders which were non-homogeneous due to the presence of included hyper-transparencies, located peripherally in the lower 1/3 of the right hemithorax; reticular opacities arranged bilaterally, images suggestive of fibrotic lesions (Figure 2).

Laboratory blood tests showed mild leukocytosis (12,590/uL) with neutrophilia (9690/uL), inflammatory syndrome (CRP = 159 mg/L, VSH = 114 mm/h), and moderate normochromic normocytic anemia (Hb = 10 g/dL); otherwise, there were no significant changes.

Arterial blood gases were taken, with the following findings: pH = 7.47; pCO_2_ = 30.1 mmHg; pO_2_ = 85.5 mmHg, HCO_3_ = 21.7 mmol/L, SO_2_ = 97% with 3 L O_2_/min (Table 1).

Antibiotic treatment (amoxicillin + clavulanic acid), pain reliever (metamizole), anticoagulant (LMWH) in prophylactic dose, antitussive treatment (codeine), and steroid therapy were initiated. Oxygen therapy with low flow 3–4 L O_2_/min through a nasal cannula was maintained. It should be mentioned that on the eighth day of hospitalization, the patient was weaned from oxygen therapy due to the peripheral blood oxygen saturation reaching values of 97% in ambient air.

**Figure 3:**
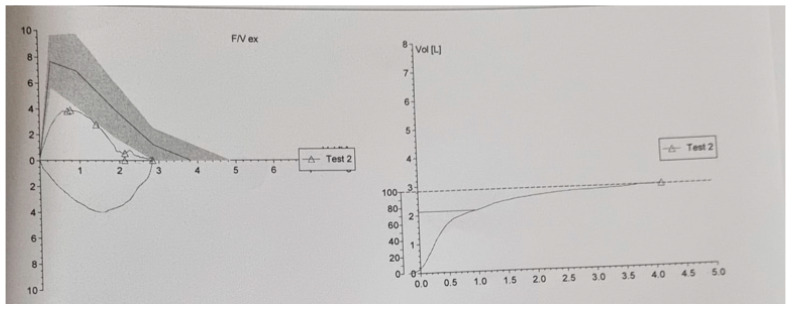
Ventilatory investigations.

A respiratory function test was performed which revealed mild restrictive ventilatory dysfunction [FVC = 74.1% (2.8 L); FEV1 = 74.7% (2.12 L); Tiffneau index = 74.73%; MEF50 = 70.7% (2.77 L)] (Table 2, Figure 3).

**Figure 4:**
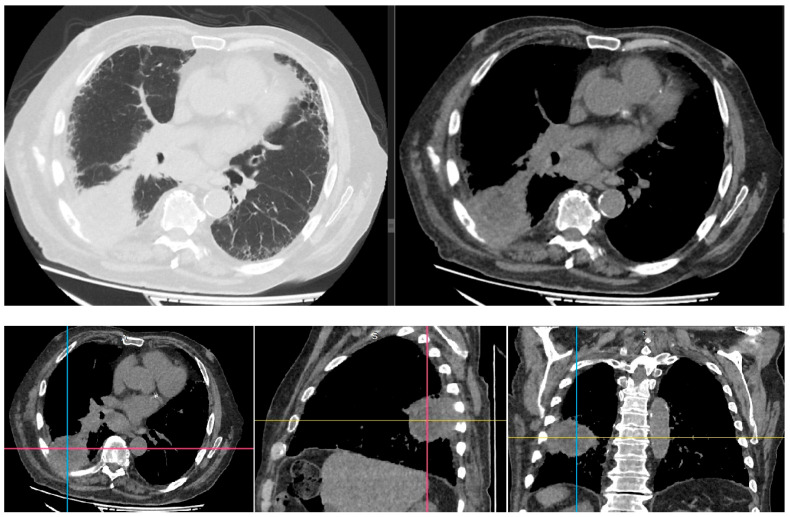
Computed tomography showing the tumor and parietal invasion.

**Figure 5:**
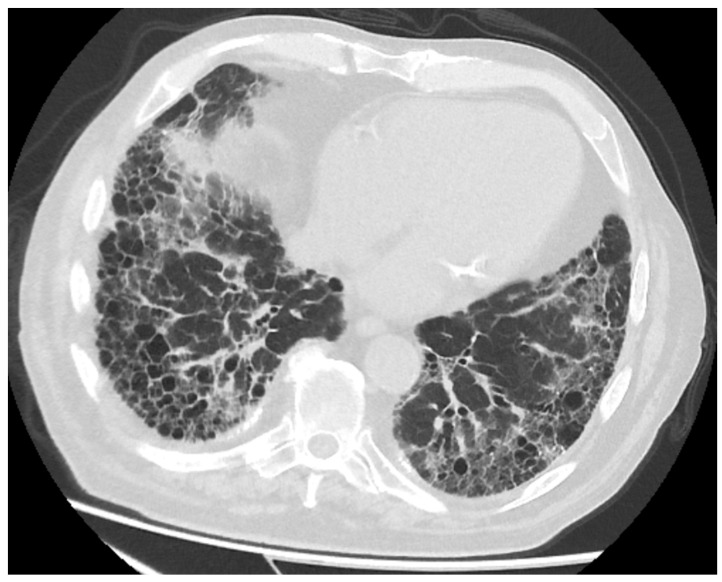
Computed tomography showing interstitial fibrosis.

Computed tomographic examination revealed a 7 cm/6 cm lung tumor situated in the right lower lobe of the lung with parietal invasion; C7 rib lysis and diffuse fibrotic interstitial changes were predominantly in the lower lobes (Figure 4 and Figure 5).

The radiology department was contacted to perform an image-guided transthoracic lung biopsy, and the decision was made to perform an ultrasound-guided biopsy due to the facile positioning of the tumor.

Three 20 mm fragments were harvested for histopathological examination and immunohistochemical tests. No postprocedural complications.

Histopathological examination established the diagnosis of invasive squamous cell lung carcinoma, G2. Perineural invasion/PN1.

Complex functional tests are performed to monitor lung function in the context of diffuse interstitial lung disease secondary to rheumatoid arthritis. Plethysmography and DLCO (Table 3) showed moderate restrictive ventilatory dysfunction with a 39.9% decrease in TLC. Normal flow resistance. Severely decreased alveolocapillary membrane gas transfer factor [FVC = 66.9% (2.57 L); FEV1 = 78% (2.23 L); Tiffneau index = 87%; MEF50 = 92% (3.6 L); TLC = 67.7% (4.83 L); RV = 76.7% (2.19 L); DLCO = 44.5% (3.76 L); KCO = 77.2% (0.92 L)].

The patient was discharged in a condition of being hemodynamically stable and respiratory signs were stable, with the following final diagnoses: right lower lobe neoplasm (G2 invasive squamous cell carcinoma); C7 right posterior costal arch rib fracture in the context of tumor invasion, rheumatoid arthritis with diffuse interstitial lung involvement, acute hypoxemic respiratory failure (currently remitted). He is referred to the oncology department for further investigations and treatment.

## 4. Discussion

Lim XR et al. conducted a cohort study observing the incidence and types of neoplasia among patients with rheumatoid arthritis. They concluded that there is an increasing trend of malignancy in rheumatoid arthritis patients compared to the general population. Namely, there is an increased risk of lymphomas in all patients with rheumatoid arthritis, an increased risk of lung neoplasm in male patients, and an increased risk of cervical neoplasm in female patients [4].

Solomon et al. carried out an observational cohort study of patients with rheumatoid arthritis who received antirheumatic medication and the potential risk of cancer due to the medication received. They found that the risk of cancer was higher for patients who received methotrexate compared to those who received biologic therapies and TNF-alpha antagonists [5]. Another study showed that rheumatoid arthritis patients treated with methotrexate had a higher incidence of neoplasms (melanoma, non-Hodgkins lymphoma, and lung cancer) compared to the general population [6].

Liu X et al. studied the characteristics of lung cancer among patients with rheumatoid arthritis and observed that patients with lung neoplasm and rheumatoid arthritis were diagnosed at a more advanced stage and had a lower ECOG score than patients with lung cancer without connective tissue involvement [7]. According to the study, our patient was also diagnosed at a late stage, when the tumor invaded the chest wall, thus limiting therapeutic options.

Another study looked at prognosis and life expectancy among patients with lung cancer and rheumatoid arthritis and without rheumatoid arthritis, respectively. It was observed that patients with lung cancer and rheumatoid arthritis had a worse prognosis and a lower survival rate than those without rheumatoid arthritis [8].

Spagnolo et al. mention in their article risk factors contributing to the development of interstitial lung disease among patients diagnosed with rheumatoid arthritis. Among these, they list smoking, older age, male sex, and seropositive arthritis, all of which were found in our patient. Also, these patients are at high risk of infections and of aggravation of pulmonary fibrosis due to drug toxicity. The authors point out that the usual interstitial pneumonia (UIP) subtype of RA-related ILD shares several clinical and histopathological features with idiopathic pulmonary fibrosis, which would point to a link between the two [9].

Rubbert-Roth et al. raise the issue of therapy in cases of patients who are simultaneously diagnosed with rheumatoid arthritis and lung neoplasm. They draw attention to the use of immune checkpoint inhibitors as antineoplastic therapy when a pathology with an autoimmune mechanism coexists and suggest an interdisciplinary approach. Also, patients diagnosed with rheumatoid arthritis and on immunosuppressive therapy would additionally benefit from lung cancer screening [10].

Pulmonary involvement is present in approximately 60–80% of patients diagnosed with rheumatoid arthritis and is the most common extra-articular involvement. Hui Huang et al. perform a review on this issue from the point of view of a chest physician and highlight the poor prognosis of patients diagnosed with RA-related pulmonary fibrosis. The authors draw attention to the need to establish morphological type in RA-related ILD and even to start antifibrotic treatment for those with progressive fibrosing ILD. In addition to ILD, lung damage in AR can occur in the form of bronchiectasis, bronchiolitis, pleurisy, rheumatoid nodules, bronchopleural fistula pneumothorax, pulmonary hypertension, pulmonary vasculitis, pulmonary embolism, pulmonary lymphoproliferative pathologies, and cancer [11].

MTX was the first conventional DMARD for RA. However, diffuse lung disease has been reported as an adverse effect of MTX. There have been multiple described cases in the literature regarding pneumonitis during methotrexate administration. The reported prevalence of MTX pneumonitis ranges from 0.3% to about 10%. It commonly appears as a sub-acute process of cough, dyspnea and diffuse ground glass opacities or consolidation, commonly with febrile illness and aggravation over multiple weeks. Even though there are cases of delayed-onset pneumonitis, approximately 50% of cases are diagnosed relatively quickly, within a year of starting methotrexate treatment [12]. Similar to the Spagnolo et al. study, Hui Huang describes risk factors for the development of pulmonary fibrosis among patients treated with methotrexate and includes among them previous use of DMARDs, hypoalbuminemia, and HLA-A31:01 haplotype. Older age, diabetes, and severe lung involvement of RA are also risk factors [11].

However, some authors are of the opinion that methotrexate should not be banned as a treatment for interstitial lung disease.

Pierre-Antoine Juge et al. conducted a case–control study among 410 patients with ILD associated with RA who were given methotrexate and 673 patients with RA without ILD. ILD-specific patterns were followed by high-resolution computed tomography. MTX users were found to have later identification of changes specific to interstitial lung disease than those who had never used methotrexate. The authors conclude that methotrexate use is not associated with a higher risk of developing RA-ILD and that ILD was identified later in MTX users [13].

Similar to Lim’s study [4], in 2008, an article was published by Ritu Khurana et al.; here, they compared the incidence of lung cancer in the veteran population diagnosed with RA versus undiagnosed RA. The study is a retrospective case–control study that collected data from the Veterans Integrated Service Networks (VISN) 16 Veteran Affairs (VA) database over 6 years (1998–2004). A total of 483,721 VA patients were included and their data were analyzed. The results show a 43% higher additional risk of developing neoplastic lung disease among patients with RA [14].

In a recent (2022) population-based cohort study among 44,101 patients with rheumatoid arthritis and approximately 216,000 people from the general population, it was found that patients with RA who were ever-smokers had a risk of developing lung neoplasms that was almost seven times greater than that of the general population, with RA seropositivity being an independent risk factor [15].

It is well known that chronic inflammation increases the risk of developing neoplastic pathologies and many other diseases, such as diabetes, autoimmune, or cardiovascular diseases. The paper by Khansari et al. explains the association between oxidative stress caused by inflammation and that caused by carcinogenesis [16]. This is particularly relevant to the case described by us because the patient simultaneously associates three individual risk factors for the development of a neoplasm: smoking, chronic inflammation due to rheumatoid arthritis, and treatment for the underlying disease.

De Cock et al. ask the following question in their manuscript: why do patients with RA have a higher risk of developing some neoplasms (lymphoma, lung cancer) and a lower risk for others (colon cancer, breast cancer)? It appears that certain types of neoplasm and rheumatoid arthritis diagnoses share common risk factors, such as smoking. It is estimated that smoking is responsible for 85% of lung cancers and increases the risk of developing rheumatoid arthritis by up to 40% [17]. It is difficult to prove whether these patients, along with the one we are referring to, had environmental risk factors or a certain genetic susceptibility to simultaneously develop these pathologies.

Some studies have failed to identify an association between methotrexate use and increased cancer risk. Mamtani et al. examine the risk of recurrence among women with breast cancer who have used methotrexate, thiopurine, or anti-TNF therapy [18]. The authors conclude that there was no statistically significant increase in the risk of recurrence among patients who used methotrexate, but they could not rule out a two-fold increased risk of recurrence among those who received anti-TNF therapy.

C. Bologna et al. conducted a study in 1997 comparing 426 patients with RA treated with MTX with 420 patients with RA not receiving MTX. From the first group, eight cases of cancer were identified and from the second group, six cases were found to have neoplasms [19]. The low number of patients with cancer in the methotrexate-treated group, the absence of an increased incidence compared to the control group, and the pluri-localization of neoplasms led the authors to conclude that they did not identify methotrexate treatment as a risk factor for carcinogenesis.

A hypothesis concerning dose-dependent adverse effects has been put forward by Solomon et al., in 2020, when they studied the adverse events occurring in known patients with cardiovascular disease, diabetes, or metabolic syndrome receiving low-dose methotrexate (LD-MTX) vs. placebo [20]. It appeared that patients receiving LD-MTX had an increased risk for any adverse event, including skin cancer, but good tolerability when given folic acid.

## 5. Conclusions

According to the literature data, rheumatoid arthritis is an individual risk factor for cancer, especially lung cancer, alongside smoking and DMARDs. There are studies which stipulate that DMARDs used to treat rheumatoid arthritis increases the risk of cancer and interstitial lung disease, but they are not always conclusive and are met by contradictions from other research papers. Drug dosage, genetic and environmental risk factors, and chronic inflammatory diseases should be considered when discussing the carcinogenic risk of a certain entity. Chest pain interpreted by the patient as rheumatic pain delayed the diagnosis of lung cancer, and the patient presented quite late to the hospital when respiratory failure set in. In our patient’s case, the tumor location and the large size allowed a relatively easy diagnosis of the neoplasm and we managed to refer him to an oncology service for further multidisciplinary treatment. Unfortunately, we lost contact with the patient after discharge and have no follow-up data to report. Further screening for lung cancer is necessary among patients with rheumatoid arthritis, as they present with multiple oncogenic risks—the disease itself, genetic traits, and the treatment. Moreover, smoking and RA seropositivity are independent risk factors for developing lung cancer in these patients. Caution should be exercised among physicians regarding patients diagnosed with RA, as they are at risk of developing interstitial lung diseases and many more pulmonary complications.

## Figures and Tables

**Table 1:** Admission arterial blood gases.

ACID/BASE	37 °C		OXYGEN 37 °C		
pH	7.476		BO_2_	15.5	mL/dL
pCO_2_	30.1	mmHg	ctO_2_	15.3	mL/dL
pO_2_	85.5	mmHg	ELECTROLITES		
HCO_3_^− act^	21.7	mmol/L	Na+	128.2	mmol/L
HCO_3_^− std^	23.6	mmol/L	K+	3.22	mmol/L
BE(B)	−1.1	mmol/L	Ca++	0.65	mmol/L
BE(ecf)	−1.9	mmol/L	Ca++(7.4)	0.67	mmol/L
CtCO_2_	22.6	mmol/L	Cl−	100	mmol/L
CO-OXIMETRY			AnGap	9.7	mmol/L
Hct	33	%	mOsm	261.3	mmol/kg
tHb	11.2	g/dL	METABOLITES		
sO_2_	96.9	%	Glu	89	mg/dL
FO_2_Hb	96.5	%	Lac	1.75	mmol/L
FCOHb	0.1	%	pAtm	760	mmHg
FMetHb	0.3				
FHHb	3.1				

**Table 2:** Ventilatory investigations.

		Pred	Pred LL	Test	Test 2	% (Test)	
VC MAX	L	3.97	3.05		2.84	71,6	%
FVC	L	3.83	2.83		2.84	74.1	%
FEV1	L	2.84	2.01		2.12	74.7	%
FEV1%M	%	72.81	61.02		74.73	102.6	%
FEV1%F	%	72.81	61.02		74.73	102.6	%
PEF	L/s	7.64	5.65		3.90	51.1	%
MEF50	L/s	3.92	1.74		2.77	70.7	%
MEF50%	%	102.18	102.18		97.37	95.3	%
FET	sec				4.19		
VBEex	L				0.14		
VBe%FV	%				4.99		
Level date				2 August 2023	2 August 2023		
Level time				11:57 A.M.	11:58 A.M.		

**Table 3:** Plethysmography and DLCO.

Plethysmography					
R tot	[kPa*s/L]	0.30	0.30	0.27	91.4
SG tot	[1/(kPa*s)]	0.85	0.85	1.03	120.7
FRCpleth	[L]	3.80	2.81	3.03	79.7
RV	[L]	2.86	2.19	2.19	76.7
TLC	[L]	7.14	5.99	4.83	67.7
VC IN	[L]	3.97	3.05	2.64	66.5
FVC	[L]	3.83	2.83	2.57	66.9
FEV1	[L]	2.84	2.01	2.23	78.4
FEV1% VC MAX	[%]	72.81	61.05	84.44	116.0
FEV1% FVC	[%]			86.88	
PEF	[L/s]	7.64	5.65	4.74	62.0
MEF 50	[L/s]	3.92	1.75	3.60	92.0
MEF 50% FVC	[%]	102.18	102.18	140.41	137.4
FET	[s]			3.20	
FEV6	[L]				
Vbackextrapolation ex	[L]			0.12	
Vbackextrapol.% FVC	[%]			4.68	
DLCO					
DLCO SB	[mmol/min/kPa]	8.47	6.15	3.76	44.5
DLCO/VA	[mmol/min/kPa/L]	1.19	0.81	0.92	77.2
Hb	[g/100 mL]			14.60	
DLCOc SB	[mmol/min/kPa]	8.47	6.15	3.76	44.5
DLCOc/VA	[mmol/min/kPa/L]	1.19	0.81	0.92	77.2
TA	[s]			10.90	
VA	[L]	6.99	6.99	4.11	58.8
VIN	[L]	3.97	3.05	2.62	66.1
Discard vol	[L]			0.75	
Sample vol	[L]			0.60	
Insp. time	[s]			0.50	
Exp. time	[s]			0.60	
TLC-SB	[L]	7.14	5.99	4.29	60.1
FRC-SB	[L]	3.80	2.81	2.50	65.8
RV-SB	[L]	2.86	2.19	1.67	58.2

## Data Availability

Data used to support the findings of this study are available from the corresponding author upon request.

## References

[1] Kourilovitch M., Galarza-Maldonado C., Ortiz-Prado E. (2014). Diagnosis and classification of rheumatoid arthritis. J. Autoimmun..

[2] Zhang Y., Lin J., You Z., Tu H., He P., Li J., Gao R., Liu Z., Xi Z., Li Z. (2022). Cancer risks in rheumatoid arthritis patients who received immunosuppressive therapies: Will immunosuppressants work?. Front. Immunol..

[3] The Global Cancer Observatory—March, 2020. https://gco.iarc.fr/today/data/factsheets/populations/900-world-fact-sheets.pdf.

[4] Lim X.R., Xiang W., Tan J.W.L., Koh L.W., Lian T.Y., Leong K.P., Koh E.T. (2019). TTSH Rheumatoid Arthritis Study Group. Incidence and patterns of malignancies in a multi-ethnic cohort of rheumatoid arthritis patients. Int. J. Rheum. Dis..

[5] Solomon D.H., Kremer J.M., Fisher M., Curtis J.R., Furer V., Harrold L.R., Hochberg M.C., Reed G., Tsao P., Greenberg J.D. (2014). Comparative cancer risk associated with methotrexate, other non-biologic and biologic disease-modifying anti-rheumatic drugs. Semin. Arthritis Rheum..

[6] Buchbinder R., Barber M., Heuzenroeder L., Wluka A.E., Giles G., Hall S., Harkness A., Lewis D., Littlejohn G., Miller M.H. (2008). Incidence of melanoma and other malignancies among rheumatoid arthritis patients treated with methotrexate. Arthritis Rheum..

[7] Liu X., Xu Y., Zhou Q., Chen M., Liang H., Zhao J., Zhong W., Wang M. (2018). Clinicopathological features of lung cancer in patients with rheumatoid arthritis. J. Thorac. Dis..

[8] Zhang L., Zhao Q., Yuan F., Liu M. (2020). Lung cancer in patients with and without rheumatoid arthritis: A propensity score-matched survival analysis cohort study. Thorac. Cancer.

[9] Spagnolo P., Lee J.S., Sverzellati N., Rossi G., Cottin V. (2018). The Lung in Rheumatoid Arthritis: Focus on Interstitial Lung Disease. Arthritis Rheumatol..

[10] Rubbert-Roth A., Zander T., Kneitz C., Baerwald C., Wirtz H., Witt C. (2016). Lungenkarzinom und rheumatoide Arthritis. Eine interdisziplinäre Herausforderung [Lung cancer and rheumatoid arthritis. An interdisciplinary challenge]. Z. Rheumatol..

[11] Huang H., Chen R., Shao C., Xu Z., Wolters P.J. (2023). Diffuse lung involvement in rheumatoid arthritis: A respiratory physician’s perspective. Chin. Med. J. (Engl.).

[12] Kremer J.M., Alarcón G.S., Weinblatt M.E., Kaymakcian M.V., Macaluso M., Cannon G.W., Palmer W.R., Sundy J.S., St. Clair E.W., Alexander R.W. (1997). Clinical, laboratory, radiographic, and histopathologic features of methotrexate-associated lung injury in patients with rheumatoid arthritis: A multicenter study with literature review. Arthritis Rheum..

[13] Juge P.A., Lee J.S., Lau J., Kawano-Dourado L., Rojas Serrano J., Sebastiani M., Koduri G., Matteson E., Bonfiglioli K., Sawamura M. (2021). Methotrexate and rheumatoid arthritis associated interstitial lung disease. Eur. Respir. J..

[14] Khurana R., Wolf R., Berney S., Caldito G., Hayat S., Berney S.M. (2008). Risk of development of lung cancer is increased in patients with rheumatoid arthritis: A large case control study in US veterans. J. Rheumatol..

[15] Chatzidionysiou K., di Giuseppe D., Soderling J., Catrina A., Askling J. (2022). Risk of lung cancer in rheumatoid arthritis and in relation to autoantibody positivity and smoking. RMD Open.

[16] Khansari N., Shakiba Y., Mahmoudi M. (2009). Chronic inflammation and oxidative stress as a major cause of age-related diseases and cancer. Recent Pat. Inflamm. Allergy Drug Discov..

[17] De Cock D., Hyrich K. (2018). Malignancy and rheumatoid arthritis: Epidemiology, risk factors and management. Best Pract. Res. Clin. Rheumatol..

[18] Mamtani R., Clark A.S., Scott F.I., Brensinger C.M., Boursi B., Chen L., Xie F., Yun H., Osterman M.T., Curtis J.R. (2016). Association Between Breast Cancer Recurrence and Immunosuppression in Rheumatoid Arthritis and Inflammatory Bowel Disease: A Cohort Study. Arthritis Rheumatol..

[19] Bologna C., Picot M.C., Jorgensen C., Viu P., Verdier R., Sany J. (1997). Study of eight cases of cancer in 426 rheumatoid arthritis patients treated with methotrexate. Ann. Rheum. Dis..

[20] Solomon D.H., Glynn R.J., Karlson E.W., Lu F., Corrigan C., Colls J., Xu C., MacFadyen J., Barbhaiya M., Berliner N. (2020). Adverse Effects of Low-Dose Methotrexate: A Randomized Trial. Ann. Intern. Med..

